# The complete mitochondrial genome of *Salmo trutta fario* Linnaeus (Salmoniformes, Salmoninae)

**DOI:** 10.1080/23802359.2016.1192501

**Published:** 2016-07-10

**Authors:** Fugui Li, Yiwen Sun, Jie Chen, Xiayun Jiang, Shuming Zou

**Affiliations:** College of Fisheries and Life Science, Key Laboratory of Freshwater Aquatic Genetic Resources, Shanghai Ocean University, Shanghai, China

**Keywords:** Genome, mitochondrial DNA, Salmonidae, *Salmo trutta fario* Linnaeus

## Abstract

The complete mitochondrial genome of *Salmo trutta fario* Linnaeus was obtained by PCR amplification and sequencing. It was 16,684 bp in length and contained 13 protein-coding genes, 22 tRNA genes, 2 rRNA and a control region. The arrangement of genes was identical to that of most other bony fishes. Nucleotide identity between *Salmo trutta fario* Linnaeus and 44 other Salmonidae species across nucleotide sequence of 12 protein-coding genes on the heavy strand was 90.0–97.5%.

*Salmo trutta fario* Linnaeus, is a geographical isolation-induced subspecies of *Salmo trutta*, with a distribution limited to the Tibet Yadong River of China (Zhang & Wang 1962). It has been listed as a critically endangered species and secondary protection wildlife by Tibet due to over commercial harvesting and trade. However, little genetic information is available on this subspecies. Hence, the complete mitochondrial genome sequence and its phylogenetic positioning within the Salmonidae are likely to support conservation and management planning of this species.

*Salmo trutta fario* L. was captured from the Yadong River in China (27°23′N, 88°52′E). The specimen is stored in China Ichthyic Culture Museum with accession number of 201208-34. Firstly, eight sets of primer pairs were designed from the mitochondrial conservative region of 16S rRNA*, CO I*, *CO II*, *CO III*, *ND4*, *ND5*, *Cytb* and *Control region* genes based on multiple sequence alignment of complete mtDNAs of other Salmonidae fish species, then partial sequences of them were amplified and sequenced. Based on these sequences, eight primer pairs were used to amplify contiguous and overlapping segments between the genes (Li et al. 2015).

The complete mtDNA sequence of *Salmo trutta fario* L. was 16,684 bp long (GenBank accession number KT 634053). It contained 13 protein-coding genes, 22 tRNA genes, 2 rRNA genes and a control region. The arrangement of the genes was identical to that of other vertebrates (Lashari et al. 2015; Shikano et al. 2016). The overall base composition was 25.9% for T, 29.5% for C , 28.1% for A and 16.5% for G.

To investigate the phylogenetic position of *Salmo trutta fario* L., phylogenetic tree were constructed by the Bayesian and maximum-likelihood methods with 12 heavy-strand protein-coding genes (10,910 bp) under GTR + I + U model. *Salmo trutta fario* L. was phylogenetically positioned with other *Salmo trutta*, showing a clear divergence from them (Figure 1). Nucleotide identity between *Salmo trutta fario* Linnaeus and 44 other Salmonidae species across nucleotide sequence of 12 protein-coding genes on the heavy strand was 90.0–97.5%.

**Figure 1.**
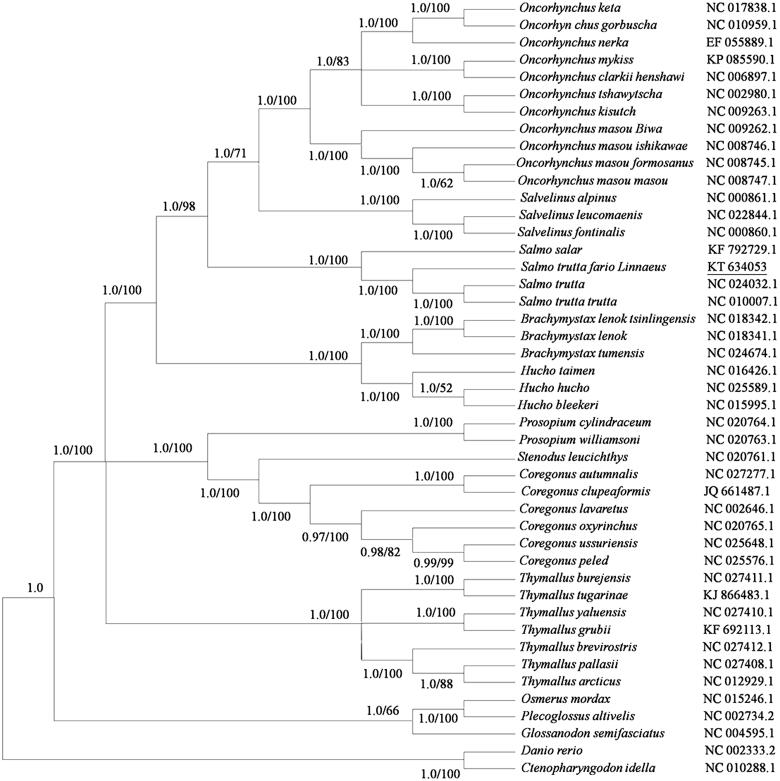
Phylogenetic tree of Salmonidae by the Bayesian and Maximum-likelihood methods based on the concatenated nucleotide sequence of 12 protein-coding genes on the heavy strand. Values less than 0.95 of Bayesian posterior probabilities and 50% of bootstrap values were omitted.

## References

[CIT0001] Lashari P, Laghari MY, Xu P, Zhao Z, Jiang L, Narejo NT, Deng Y, Sun X, Zhang Y. 2015 Complete mitochondrial genome of the freshwater catfish *Rita rita* (Siluriformes, Bagridae). Mitochondrial DNA. 26:817–818. 24409859 10.3109/19401736.2013.855908

[CIT0002] Li W, Gong S, Hua L, Ge Y, Wang F, Hou F. 2015 Complete mitochondrial genome sequence for the Malayan Pangolin *Manis javanica* (Pholidota, Manidae). Conservation Genet Resour. 7:685–687.

[CIT0003] Shikano T, Guo B, Vukić J, Šanda R, Merilä J. 2016 Complete mitochondrial genome of the greek nine-spined stickleback *Pungitius hellenicus* (Gasterosteiformes, Gasterosteidae). Mitochondrial DNA. 1:66–67. 10.1080/23802359.2015.1137826 PMC7800052 33473410

[CIT0004] Zhang CL, Wang WB. 1962 A preliminary report on the fishes from Tibet. Acta Zoologica Sinica. 14:529–536.

